# Glutamine synthetase is necessary for sarcoma adaptation to glutamine deprivation and tumor growth

**DOI:** 10.1038/s41389-019-0129-z

**Published:** 2019-02-26

**Authors:** Sameer H. Issaq, Arnulfo Mendoza, Stephen D. Fox, Lee J. Helman

**Affiliations:** 1 Pediatric Oncology Branch, National Cancer Institute, National Institutes of Health, Bethesda, MD USA; 2 Protein Characterization Laboratory, Frederick National Laboratory for Cancer Research, Leidos Biomedical Research, Inc., Frederick, MD USA; 3 Departments of Pediatrics and Medicine, Keck School of Medicine, University of Southern California, Los Angeles, CA USA; 4 Children’s Center for Cancer and Blood Diseases, Children’s Hospital Los Angeles, Los Angeles, CA USA

## Abstract

Despite a growing body of knowledge about the genomic landscape and molecular pathogenesis of sarcomas, translation of basic discoveries into targeted therapies and significant clinical gains has remained elusive. Renewed interest in altered metabolic properties of cancer cells has led to an exploration of targeting metabolic dependencies as a novel therapeutic strategy. In this study, we have characterized the dependency of human pediatric sarcoma cells on key metabolic substrates and identified a mechanism of adaptation to metabolic stress by examining proliferation and bioenergetic properties of rhabdomyosarcoma and Ewing sarcoma cells under varying concentrations of glucose and glutamine. While all cell lines tested were completely growth-inhibited by lack of glucose, cells adapted to glutamine deprivation, and restored proliferation following an initial period of reduced growth. We show that expression of glutamine synthetase (GS), the enzyme responsible for de novo glutamine synthesis, increased during glutamine deprivation, and that pharmacological or shRNA-mediated GS inhibition abolished proliferation of glutamine-deprived cells, while having no effect on cells grown under normal culture conditions. Moreover, the GS substrates and glutamine precursors glutamate and ammonia restored proliferation of glutamine-deprived cells in a GS-dependent manner, further emphasizing the necessity of GS for adaptation to glutamine stress. Furthermore, pharmacological and shRNA-mediated GS inhibition significantly reduced orthotopic xenograft tumor growth. We also show that glutamine supports sarcoma nucleotide biosynthesis and optimal mitochondrial bioenergetics. Our findings demonstrate that GS mediates proliferation of glutamine-deprived pediatric sarcomas, and suggest that targeting metabolic dependencies of sarcomas should be further investigated as a potential therapeutic strategy.

## Introduction

Sarcomas comprise a diverse group of mesenchymal malignancies that are derived from connective and soft tissues, including muscle, bone, and cartilage. Sarcomas affect approximately 200,000 individuals worldwide each year and represent a higher percentage of overall cancer morbidity and mortality in children and young adults than in adults^1,2^. Pediatric sarcomas, including rhabdomyosarcoma (RMS) and Ewing sarcoma (ES), account for almost 21% of all pediatric solid malignancies and constitute a significant mortality burden of about 13% of cancer-related deaths in patients 0–19 years of age^3,4^. Rhabdomyosarcoma is the most common soft tissue sarcoma of childhood and adolescence. RMS tumors express skeletal muscle markers, but resemble histologically aberrant muscle differentiation states. They often originate in or near muscle beds, but can arise virtually anywhere in the body, including sites lacking skeletal muscle, such as the biliary and genitourinary tract^5,6^. Ewing sarcoma is a highly aggressive bone and soft tissue malignancy that primarily affects children and adolescents in the second decade of life. ES is the second-most common pediatric malignant bone tumor^7–9^.

Despite a growing body of knowledge about the genomic landscape and molecular pathogenesis of RMS and ES, the successful translation of basic discoveries into molecularly targeted therapies and significant clinical gains has remained elusive^8,10,11^. There are relatively few recurrent genetic mutations driving tumorigenesis for the majority of pediatric sarcomas, and ES tumors possess one of the lowest somatic mutation rates among all human cancers (0.15 mutations/megabase)^8,11,12^. Rather, approximately one-third of all sarcomas are driven by chimeric transcription factors, which are the result of well-defined chromosomal translocations^1,11^. Indeed, this is especially true of ES and the most aggressive form of RMS. These oncogenic, chimeric transcription factors are extremely challenging drug targets due to disordered protein structure and lack of intrinsic enzymatic activity^8,12^.

Reflecting the lack of molecularly targeted therapies, treatment for RMS and ES similarly includes a combination of conventional cytotoxic chemotherapeutic agents, and local control of the primary tumor with surgery and/or radiation. While this aggressive, multimodal treatment approach has improved long-term survival rates for patients with localized disease to around 70%, patients with metastatic or recurrent disease have a very poor 5-year survival rate of less than 20–30%^3,6–11,13^. Furthermore, the acute and long-term toxicities associated with exposure to current therapeutic regimens at such a young age are considerable, and those who do survive RMS and ES face a lifetime of significant treatment-related effects, including profound functional and cosmetic deficits, organ toxicities, secondary malignancies, and shortened life expectancies^3,6,9^. Therefore, novel therapeutic strategies for pediatric sarcomas are critically important, not only to increase survival in patients with metastatic or relapsed disease, but to continue to improve survival of patients with localized disease, as well as to decrease the acute and chronic toxicities associated with current therapies^2,3,10^.

Renewed interest in the metabolic properties of cancer cells has led to an exploration of targeting specific metabolic dependencies as a viable therapeutic strategy^14,15^. Many signaling pathways affected by genetic events in cancer, as well as the tumor microenvironment, can significantly alter cellular metabolism to meet the increased biosynthetic and energy demands necessary to support cancer cell survival and proliferation^14,15^. As such, changes in cellular metabolism are now recognized as a crucial hallmark of cancer^16^. Cancer cells exhibit a metabolic phenotype known as aerobic glycolysis, or the Warburg effect, which is characterized by increased glycolysis, even in the presence of sufficient oxygen to support mitochondrial oxidative phosphorylation^15,17^. Increased glucose uptake, which often accompanies aerobic glycolysis, can be visualized in patient tumors using ^18^F-deoxyglucose positron emission tomography (FDG–PET) imaging. FDG–PET is used clinically as a staging tool for several diverse types of cancers, including pediatric sarcomas like RMS and ES, where it is especially useful in the identification of skeletal and lymph node metastases and unknown primary sites, and has been reported to be a predictor of patient outcome and disease progression, and to correlate with histologic response to therapy^15,18–22^. Another major change in the metabolic program of many cancer cells is the alteration of glutamine metabolism^23^. Glutamine is the most abundant amino acid in serum, and proliferating cells metabolize glutamine in multiple pathways supporting bioenergetics and biosynthesis^15,23–25^. It is the major source of nitrogen for nucleotide and amino acid synthesis, and also has an important role in replenishing intermediates of the TCA cycle (anaplerosis)^15,23,24^. Multiple studies investigating glutamine deprivation or inhibition of glutamine catabolism have identified a dependence of certain cancer cells on glutamine^15,23,24^. However, not all cancer cells need an exogenous supply of glutamine, and resistance to glutamine deprivation has been associated with de novo glutamine synthesis^26,27^. The importance of glutamine metabolism in RMS and ES tumorigenesis has not been well-characterized.

Here, we have characterized the dependency of human pediatric sarcoma cells on key metabolic substrates and identified a mechanism of adaptation to metabolic stress by examining cell proliferation and bioenergetic properties under varying concentrations of glucose and glutamine. While all cell lines were completely growth-inhibited by lack of glucose, cells were able to adapt to glutamine deprivation and restore proliferation following an initial period of reduced growth. We show that expression of glutamine synthetase (GS), the enzyme responsible for de novo glutamine synthesis, increased during glutamine deprivation, and that pharmacological or shRNA-mediated inhibition of GS abolished the ability of glutamine-deprived cells to proliferate, while having no effect on cells grown under normal culture conditions. Furthermore, pharmacological and shRNA-mediated inhibition of GS significantly reduced orthotopic xenograft tumor growth. We also show that glutamine supports sarcoma nucleotide biosynthesis and optimal mitochondrial bioenergetics. Our findings suggest that GS mediates proliferation of glutamine-deprived pediatric sarcoma cells, and that targeting metabolic dependencies may represent a novel therapeutic strategy for the treatment of sarcomas.

## Results

### Human sarcoma cells can adapt to glutamine deprivation

**Fig. 1:**
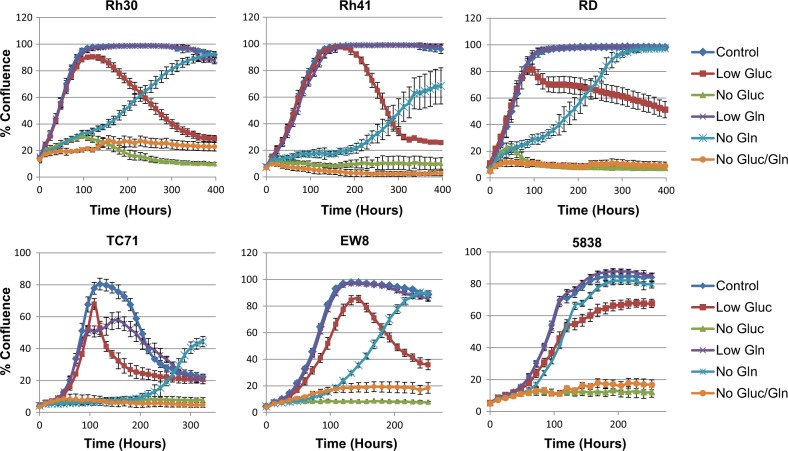
Metabolic substrate utilization of human sarcoma cell lines. Cellular proliferation was monitored in an IncuCyte FLR. Cell lines were assayed in RPMI 1640 media supplemented with 10% dialyzed FBS and the indicated concentrations of glucose and glutamine. Control (11 mM glucose, 2 mM glutamine), Low Gluc (low glucose, 1.1 mM), No Gluc (no glucose), Low Gln (low glutamine, 0.2 mM), No Gln (no glutamine), and No Gluc/Gln (no glucose/glutamine). Data represent the mean ± SD of a representative experiment

Despite a growing body of knowledge about the genomic landscape and molecular pathogenesis of sarcomas, metabolic dependencies have not been well-characterized. To examine the dependency of pediatric sarcomas on the primary metabolic nutrients glucose and glutamine, we monitored the proliferation of six human RMS (Rh30, Rh41, and RD) and ES (TC71, EW8, and 5838) cell lines grown in media supplemented with various concentrations of glucose and glutamine using an IncuCyte Live-Cell Analysis System. This system allowed us to visually follow proliferation continuously over an extended period of time, in contrast to other methods that require terminal analysis at a limited number of time points. As expected, all cell lines cultured in media lacking both glucose and glutamine did not proliferate (Fig. 1). Similarly, cells cultured in glucose-free media did not proliferate. Interestingly, cell lines cultured in glutamine-free media displayed an initial period of growth inhibition lasting approximately 2–8 days, depending on the cell line, but eventually began to proliferate (Fig. 1). Proliferation was mostly unaffected by tenfold reduced concentrations (compared to standard culture conditions) of glucose or glutamine (Fig. 1).

To further examine proliferation of glutamine-deprived sarcomas, three sarcoma cell lines were split into two flasks each and passaged in parallel, either in the presence or absence of glutamine. Following 2 weeks of continuous passaging, proliferation was assayed in media with or without glutamine, generating four groups (see schematic in Supplementary Figure S1a): Gln +/+ (passaged with glutamine, assayed with glutamine), Gln +/− (passaged with glutamine, assayed without glutamine), Gln −/+ (passaged without glutamine, assayed with glutamine), Gln −/− (passaged without glutamine, assayed without glutamine). Consistent with our earlier findings, cells continuously passaged in Control media but assayed in glutamine-free media (Gln +/−) displayed a significant initial period of growth inhibition compared to Gln +/+ cells, which varied in duration by cell line (Supplementary Figure S1b). Re-introduction of glutamine to cells continuously passaged in glutamine-free media (Gln −/+) was able to rescue proliferation to rates similar to Gln +/+ cells for all cell lines tested (Supplementary Figure S1b). Furthermore, TC71 and EW8 cells continuously passaged in glutamine-free media had an increased rate of proliferation compared to cells continuously passaged in Control media, when assayed in glutamine-free media (Gln −/− vs. Gln +/−). Taken together, these findings suggest that sarcomas can adapt to glutamine deprivation, but glutamine is necessary for maximal proliferation.

### Glutamine deprivation increases GS protein expression in sarcomas

**Fig. 2:**
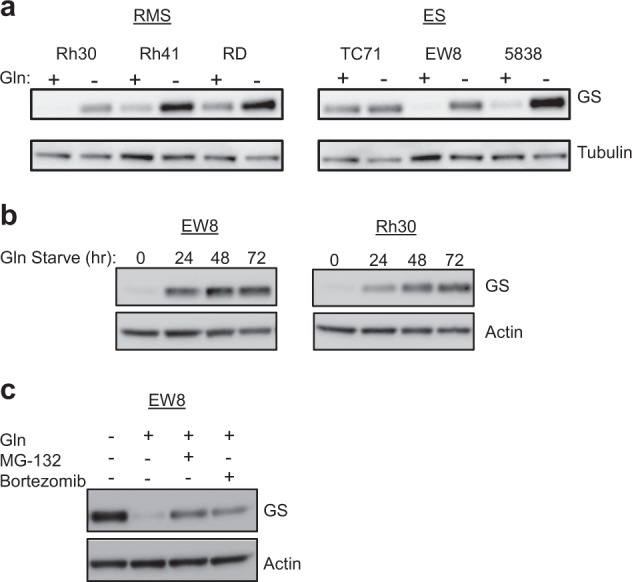
Glutamine deprivation induces glutamine synthetase protein expression. **a** Immunoblot analysis of glutamine synthetase (GS) in rhabdomyosarcoma (RMS) and Ewing sarcoma (ES) cell lines grown in the presence or absence of glutamine (Gln). Tubulin was used as a loading control. **b** Immunoblot analysis of GS in cells grown in the presence or absence of glutamine for the indicated times. Actin was used as a loading control. **c** Immunoblot analysis of GS in cells grown in the presence or absence of glutamine, MG-132 (10 μM), and Bortezomib (1 μM), as indicated. Actin was used as a loading control

Our observation that sarcomas were able to adapt and proliferate under glutamine deprivation raised the possibility that the cells may be synthesizing glutamine de novo to make up for the lack of exogenous glutamine, which depends on expression of the enzyme GS^28–30^. Increased de novo glutamine synthesis and GS expression has been reported previously in response to glutamine starvation^27,31,32^. To determine whether glutamine deprivation led to increased GS protein expression in pediatric sarcomas, we performed immunoblot analysis of GS in RMS and ES cell lines continuously passaged in the presence or absence of glutamine for at least 1 week. As shown in Fig. 2a, all six cell lines tested exhibited a clear increase in GS protein expression when cultured in glutamine-free media compared to parental cells grown in standard culture media containing glutamine, consistent with previous reports^27,31,32^.

To evaluate the timing of increased GS protein expression, a timecourse study was performed on EW8 and Rh30 cells. Parental cells were switched from standard culture media containing glutamine to glutamine-free media and collected for immunoblot analysis following 0, 24, 48, and 72 h of glutamine deprivation. As shown in Fig. 2b, GS protein expression significantly increased in both cell lines from nearly undetectable levels during the first 24 h of glutamine deprivation, and plateaued between 48 and 72 h of glutamine starvation.

### Sarcoma GS protein expression is post-translationally regulated

Recently, Nguyen et al.^33^ described a post-translational mechanism of regulation of GS protein degradation involving ubiquitination and subsequent proteosomal degradation of GS in the presence of glutamine. To determine whether GS protein stability is regulated at the post-translational level in pediatric sarcomas, we performed immunoblot analysis of GS protein expression in EW8 cells grown in the absence or presence of glutamine and treated with or without the proteosome inhibitors MG132 or Bortezomib. As shown in Fig. 2c, the addition of glutamine to glutamine-deprived EW8 cells led to an almost complete reduction of GS protein level following overnight incubation, however, this effect was blocked by the simultaneous addition of either of the proteosome inhibitors MG132 or Bortezomib at previously published concentrations^33^. These findings are consistent with the previous study^33^ and suggest that sarcoma GS protein expression is controlled, at least in part, by post-translational regulation, demonstrating the reversibility of GS upregulation and the plasticity of these cells to glutamine deprivation.

### GS expression and activity is necessary for proliferation of glutamine-deprived sarcomas

**Fig. 3:**
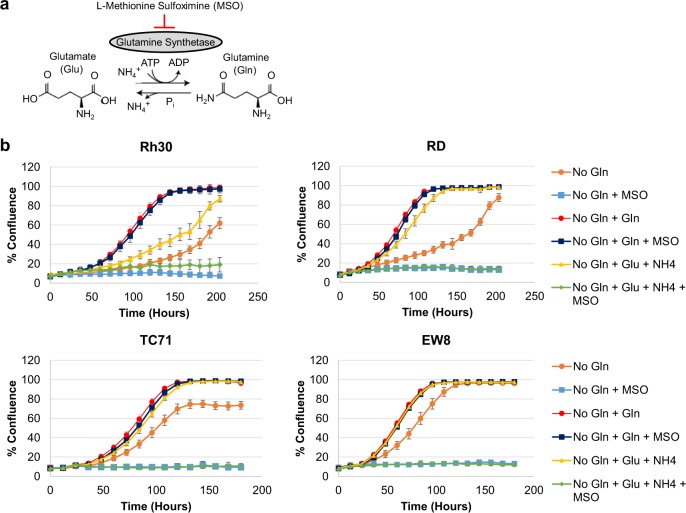
Glutamine synthetase is necessary for proliferation of glutamine-deprived sarcomas. **a** Schematic of glutamine synthetase and l-methionine sulfoximine. **b** Cellular proliferation was monitored in an IncuCyte FLR. Cell lines were assayed in RPMI 1640 media supplemented with 10% dialyzed FBS under the indicated conditions. Gln (glutamine), Glu (glutamate), NH_4_ (ammonia), MSO (l-methionine sulfoximine). Data represent the mean ± SD of a representative experiment

To determine the importance of GS activity in glutamine-deprived sarcoma proliferation, we utilized the well-characterized, irreversible GS inhibitor l-methionine sulfoximine (MSO)^29,30,34^ and the GS substrates glutamate and ammonia (see schematic in Fig. 3a). In all four cell lines tested, inhibition of GS with MSO completely abolished proliferation of glutamine-deprived cells, but had no effect on proliferation in the presence of glutamine (Fig. 3b). Furthermore, addition of the GS substrates glutamate and ammonia increased proliferation rates of all four glutamine-deprived cell lines, however, MSO treatment was able to completely block the effect of glutamate and ammonia addition (Fig. 3b). Taken together, these findings suggest that GS activity is necessary for glutamine-deprived proliferation of pediatric sarcomas, and that the effects of MSO are specific to GS inhibition.

**Fig. 4:**
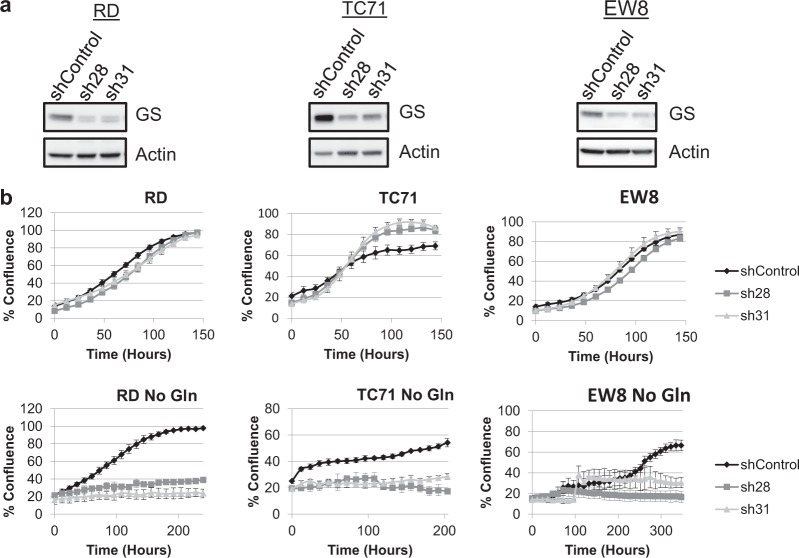
Glutamine synthetase is necessary for proliferation of glutamine-deprived sarcomas. **a** Immunoblot analysis of glutamine synthetase (GS) in the specified cell lines expressing the indicated lentiviral GS shRNAs. Actin was used as a loading control. **b** Cellular proliferation was monitored in an IncuCyte FLR. Cell lines were assayed in RPMI 1640 media supplemented with 10% dialyzed FBS in the presence or absence of glutamine, as indicated. Data represent the mean ± SD of a representative experiment

To further examine the biological significance of increased GS expression in glutamine-deprived sarcomas, stable cell lines expressing either of two distinct GS-targeting shRNAs (sh28 and sh31) or a nontargeting negative control shRNA (shControl) were generated using lentiviral particles. Knockdown of GS was confirmed by immunoblot analysis (Fig. 4a). GS knockdown by either targeting sequence in three different sarcoma cell lines had no effect on proliferation of cells grown in the presence of glutamine, however, GS knockdown severely inhibited proliferation of cells grown in glutamine-free media, compared to shControl counterparts (Fig. 4b). The similar results obtained with two distinct GS-targeting shRNAs in three different cell lines suggests that the effects seen are not due to off-target effects of the shRNAs. These findings are consistent with our results described above using the GS inhibitor MSO, confirming the necessity of GS expression and activity for proliferation of pediatric sarcomas under glutamine starvation.

### GS is necessary for sarcoma tumorigenic growth

We have shown that GS is necessary for proliferation of glutamine-deprived sarcomas in vitro, so we next examined the biological significance of GS for sarcoma tumor growth in vivo. We utilized the TC71 stable cell lines described above expressing negative control shRNA or either of two distinct GS-targeting shRNAs. We selected TC71 because of the well-characterized and consistent ability to rapidly form tumors when orthotopically injected into the gastrocnemius muscle of immunocompromised mice^35^. We also simultaneously evaluated the effect of MSO treatment, either alone or in combination with GS knockdown, on tumorigenic growth.

**Fig. 5:**
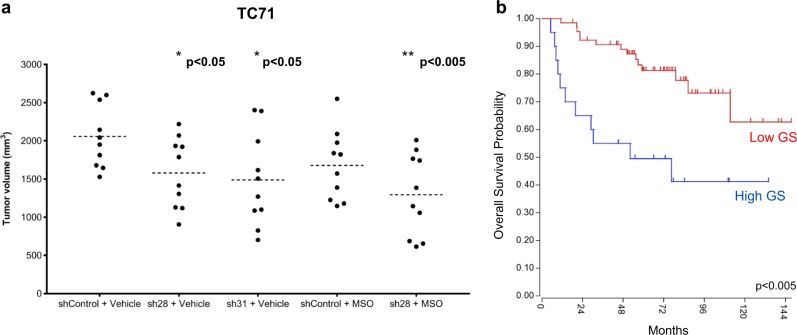
Glutamine synthetase is necessary for sarcoma tumor growth. **a** Tumor volumes at day 20 postorthotopic injection of TC71 Ewing sarcoma stable cell lines expressing the indicated shRNAs. Each data point represents the tumor volume of one mouse, and the broken line represents the average tumor volume for the group. Mice were treated with 10 mg/kg MSO or saline, IP, 3 times/week, as indicated. ^*^p < 0.05, ^**^p < 0.005 by Student *t* test, compared to shControl + Vehicle. **b** Kaplan–Meier analysis of overall survival of Ewing sarcoma patients with respect to glutamine synthetase expression, demonstrating that higher expression is correlated with worse overall survival. Analysis (Kaplan–Meier by gene expression) of the “Tumor Ewing Sarcoma (Core Transcript) - Dirksen - 85 - rma_sketch - huex10t” dataset (comprised of 85 samples) was performed using R2: Genomics Analysis and Visualization Platform (http://r2.amc.nl)

Following orthotopic injection of the aforementioned stable cell lines in immunocompromised mice, tumor growth was monitored at regular intervals. TC71 cells expressing negative control shRNA generated palpable tumors with a latency of 10 days, at which point all mice were randomized into treatment groups (10 mice/group) receiving either 10 mg/kg MSO or saline (Vehicle control) three times per week, intraperitoneally. This MSO dose and schedule was selected based on previously published data^36^. By 20 days postinjection, Vehicle control-treated shControl cells reached study endpoint tumor volumes. Figure 5a shows the resulting tumor volumes of all treatment groups at day 20. As shown in Fig. 5a, knockdown of GS with either of the two distinct GS-targeting shRNAs, sh28 or sh31, caused a statistically significant decrease (*p* < 0.05) in average tumor volume of 23% or 28%, respectively, compared to shControl tumors. Treatment of mice bearing shControl tumors with the GS inhibitor MSO led to an 18% reduction in average tumor volume compared to Vehicle-treated controls (Fig. 5a). Moreover, treatment of mice bearing GS shRNA-expressing tumors with MSO had the greatest effect on tumor growth, and led to a statistically significant 37% decrease (*p* < 0.005) in average tumor volume compared to Vehicle-treated shControl tumors (Fig. 5a). Tumor growth curves are shown in Supplementary Figure S2. Taken together, these results suggest that GS is necessary for sarcoma tumorigenic growth.

To evaluate target validation in our in vivo studies, we examined GS protein expression in GS shRNA-expressing tumors compared to shControl tumors, and we also examined changes in serum and tumor glutamate/glutamine in response to MSO treatment. Immunoblot analysis demonstrated that GS protein expression was suppressed in tumors derived from GS shRNA-expressing cells compared to shControl-expressing tumors (Supplementary Figure S3a). Interestingly, GS protein expression in GS shRNA-expressing tumors increased by an average of twofold in comparison to the pre-injection GS shRNA-expressing cell line (Supplementary Figure S3b), indicating that tumors may select for higher GS expression.

To evaluate the effects of MSO treatment in our in vivo studies we examined serum and tumor levels of glutamate and glutamine. We show that serum glutamate levels were significantly decreased (*p* < 0.005) in both MSO treatment groups of mice (shControl + MSO and GS sh28 + MSO) compared to Vehicle control-treated mice (Supplementary Figure S4a), while serum glutamine was slightly elevated, which is consistent with previous findings involving target validation of in vivo MSO treatment^36^. Additionally, although glutamine levels were not significantly altered, we show that tumor glutamate levels were dramatically increased by MSO treatment in both MSO treatment groups (shControl + MSO, *p* < 0/05 and GS sh28 + MSO, *p* < 0.005) compared to Vehicle control-treated mice (Supplementary Figure S4b), which is consistent with inhibition of GS.

To assess the importance of GS in clinical outcome, we used the R2: Genomics Analysis and Visualization Platform (http://r2.amc.nl) to examine Kaplan–Meier analysis of overall survival of Ewing sarcoma patients with respect to GS expression. As shown in Fig. 5b, higher expression of GS is correlated with worse overall survival in Ewing sarcoma patients, further supporting the importance of GS for pediatric sarcoma tumor growth.

### Glutamine is necessary for supporting optimal sarcoma mitochondrial bioenergetics and nucleotide synthesis

**Fig. 6:**
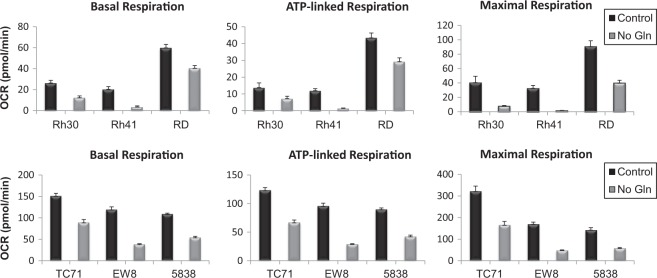
Glutamine supports optimal sarcoma mitochondrial bioenergetics. Basal respiration, ATP-linked respiration, and maximal respiration rates were determined from Seahorse bioenergetic profiles, and are represented as the mean ± SD of a representative experiment

Glutamine metabolism contributes to cellular bioenergetics and biosynthesis^15,23–25^. To determine the contribution of glutamine to sarcoma mitochondrial bioenergetics, we performed mitochondrial bioenergetic profiling of pediatric sarcoma cell lines grown in the presence or absence of glutamine using Seahorse extracellular flux analysis. As shown in Fig. 6, all six glutamine-deprived cell lines tested exhibited reduced levels of basal, ATP-linked, and maximal respiration, as well as nearly abrogated spare respiratory capacity compared to matched cell lines grown in standard glutamine-containing media, as determined from the mitochondrial bioenergetic profiles shown in Supplementary Figure S5. These findings demonstrate that glutamine significantly contributes to sarcoma mitochondrial bioenergetics.

**Fig. 7:**
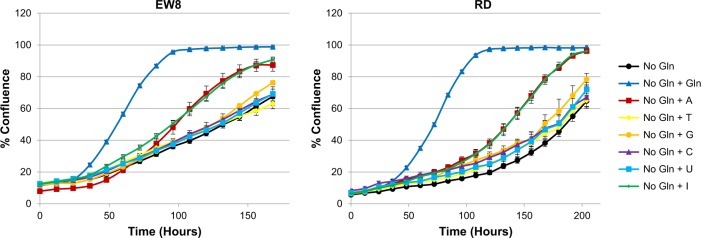
Glutamine is necessary for sarcoma nucleotide synthesis. Cellular proliferation was monitored in an IncuCyte FLR. Cell lines were assayed in glutamine-free RPMI 1640 media supplemented with 10% dialyzed FBS and either glutamine (2 mM) or nucleosides (250 μM), as indicated. Gln (glutamine), A (adenosine), T (thymidine), G (guanosine), C (cytidine), U (uridine), I (inosine). Data represent the mean ± SD of a representative experiment

Since glutamine is a precursor for purine nucleotide synthesis, we sought to determine whether the addition of nucleosides (at previously published^32^ concentrations) to glutamine-deprived sarcoma cells could rescue proliferation. As shown in Fig. 7, the addition of either the purine precursor inosine or the purine nucleoside adenosine to glutamine-deprived EW8 or RD cells caused an approximately twofold increase in proliferation, while addition of guanosine or pyrimidine nucleosides did not significantly enhance glutamine-deprived proliferation. These results suggest that glutamine contributes to sarcoma purine nucleotide synthesis to support maximal proliferation, which is consistent with previous findings^32,37^.

## Discussion

Despite a growing body of knowledge about the genomic landscape and molecular pathogenesis of sarcomas, the translation of basic discoveries into molecularly targeted therapies and clinical gains has remained elusive^8,10,11^. Renewed interest in metabolic properties of cancer cells, as well as the recognition of altered cellular metabolism as a critical hallmark of cancer, has stimulated an exploration of targeting metabolic vulnerabilities of cancer cells as a novel therapeutic strategy^14–16^. Here, we have performed the first characterization of the dependency of human pediatric sarcomas on key metabolic substrates by examining cell proliferation and bioenergetic properties of RMS and ES cells under varying concentrations of glucose and glutamine. We show that sarcoma cells were able to adapt to glutamine deprivation and restore proliferation following an initial period of growth inhibition that varied by cell line. These findings are in contrast to studies that have described a dependency on glutamine for certain cancer cells, but are consistent with other reports of proliferation independent of exogenous glutamine^15,23,24,26,27^. Given the interesting nature of our findings regarding glutamine starvation and the paucity of data on the importance of glutamine metabolism in RMS and ES, we focused on elucidating the role of glutamine in sarcoma bioenergetics and biosynthesis and identifying the mechanism of sarcoma adaptation to glutamine stress.

Our observation that sarcomas were able to adapt and proliferate under glutamine deprivation suggested that de novo glutamine synthesis may have increased, which depends on expression of the enzyme GS^28–30^. Increased de novo glutamine synthesis and GS expression has been reported previously in response to glutamine starvation, both in cancer cell lines and in tumors^27,31,32^. We show that all cell lines tested exhibited increased GS protein expression when cultured in glutamine-free media, consistent with previous reports^27,31,32^. The increase in GS protein was observed during the first 24 h of glutamine deprivation, suggesting that increased GS expression is an early response to glutamine deprivation in sarcomas that may be important for maintaining survival during periods of glutamine stress. Furthermore, we show that sarcoma GS protein stability is regulated, at least in part, at the post-translational level, which is consistent with recent work describing a feedback mechanism of GS protein degradation involving acetylation, ubiquitination and subsequent proteosomal degradation of GS in the presence of glutamine^33^.

To determine the importance of GS activity for proliferation of glutamine-deprived sarcomas, we utilized the well-characterized, irreversible GS inhibitor MSO^29,30,34^, which completely abolished proliferation of glutamine-deprived cells but had no effect on proliferation of the same cell lines grown in glutamine-containing media. Furthermore, addition of the GS substrates glutamate and ammonia to glutamine-deprived cells rescued proliferation similarly to glutamine addition, however, MSO treatment was able to completely block the effect of glutamate and ammonia addition. Taken together, these findings suggest that GS activity is necessary for glutamine-deprived proliferation of pediatric sarcomas, and that the effects of MSO are specific to GS inhibition. To further examine the biological significance of GS expression in glutamine-deprived sarcomas, we generated stable cell lines expressing GS-targeting shRNAs. We show that GS knockdown in three different sarcoma cell lines had no effect on proliferation of cells grown in glutamine-containing media, however, GS knockdown severely inhibited proliferation of the same cells when grown in glutamine-free media. These findings are consistent with our results using the GS inhibitor MSO, confirming the necessity of GS expression and function for glutamine-deprived proliferation of pediatric sarcomas.

To expand on our in vitro finding, we examined the biological significance of GS for sarcoma tumor growth in vivo using TC71 stable cell lines expressing GS-targeting shRNAs. We selected TC71 cells because of the well-characterized, consistent ability to rapidly form tumors when injected into the gastrocnemius muscle of immunocompromised mice^35^. This model closely recapitulates human Ewing sarcoma, enabling tumors to form in a physiologically relevant environment. We also simultaneously evaluated the effect of MSO treatment on tumor growth, either alone or in combination with GS knockdown. GS knockdown caused a statistically significant decrease in average tumor volume of 23 or 28% (*p* < 0.05). While MSO treatment alone led to an 18% reduction in average tumor volume, treatment of mice bearing GS shRNA-expressing tumors with MSO led to a statistically significant 37% decrease (*p* < 0.005) in average tumor volume, demonstrating additional benefit to dual targeting of GS by shRNA and MSO. Taken together, these results suggest that GS is necessary for sarcoma tumor growth, which is consistent with our in vitro findings. Clinically, we show that high GS expression is correlated with worse overall survival in Ewing sarcoma patients, further supporting the importance of GS for sarcoma tumor growth. A direct link between the genetic drivers of ES/RMS and GS expression has not been examined previously. Large-scale genomic analyses of putative transcriptional targets of sarcoma oncogenic drivers, such as the ES fusion oncoprotein EWS-FLI1, have not identified GS as a direct transcriptional target^38^. However, these findings do not exclude the possibility of GS being an indirect target of sarcoma genetic drivers.

In line with our current findings, several recent studies have demonstrated that inhibition of GS can significantly impair tumorigenic growth and metastasis in preclinical models^29,32,36,37,39^, however, there are currently no clinically approved drugs that specifically target GS^34^. The best-characterized GS inhibitor, MSO, has been largely avoided as a clinical cancer therapeutic due to central nervous system toxicity in some species, although there has been no attempt to date to establish a therapeutic index for MSO because of historical stigma^34^. Strategies to limit central nervous system toxicity of MSO and GS inhibition, such as tumor-specific drug delivery or combination therapy to lower the necessary dose, should be examined further. One such strategy involving the combination of MSO and l-asparaginase showed antitumor activity in a preclinical hepatocellular carcinoma model^36^.

Proliferating cells metabolize glutamine in multiple pathways supporting bioenergetics and biosynthesis; glutamine is the major source of nitrogen for nucleotide and amino acid synthesis, and also has an important role in the TCA cycle^15,23–25^. To determine the contribution of glutamine to sarcoma mitochondrial bioenergetics, we performed mitochondrial bioenergetic profiling of cell lines grown in the presence or absence of glutamine. All glutamine-deprived cell lines tested exhibited reduced levels of basal, ATP-linked, and maximal respiration, as well as nearly abrogated spare respiratory capacity compared to matched cell lines grown in glutamine-containing media, demonstrating that glutamine significantly contributes to maintaining optimal mitochondrial bioenergetics in sarcomas. Since glutamine is a precursor for purine nucleotide synthesis^15,23^, we examined whether the addition of nucleosides to glutamine-deprived sarcoma cells could rescue proliferation similarly to re-introduction of glutamine. We show that addition of either the purine precursor inosine or the purine nucleoside adenosine to glutamine-deprived RMS and ES cells caused an approximately two-fold increase in proliferation, suggesting that glutamine contributes to sarcoma purine nucleotide synthesis to support maximal proliferation, which is consistent with previous findings^32,37^. Therefore, we provide new insights demonstrating that glutamine metabolism fulfills bioenergetic and biosynthetic needs of pediatric sarcoma cells to maintain optimal proliferative capacity. Our findings suggest that targeting metabolic dependencies may represent a novel therapeutic strategy for the treatment of sarcomas.

## Materials and methods

### Cell culture

Human sarcoma cell lines were previously described^35,40^. All cell lines were cultured in RPMI 1640 media supplemented with 2 mM l-glutamine and 10% fetal bovine serum (FBS). For glutamine-free cell culture and certain experiments 10% dialyzed FBS was used, as indicated. All cell lines were maintained in a humidified incubator containing 5% CO_2_ at 37 °C.

### Preparation of compounds

Stock solutions of 50 mM MG-132, 10 mM Bortezomib, 5 mg/mL oligomycin, 10 mM carbonyl cyanide 4-(trifluoromethoxy)phenylhydrazone (FCCP), 1 mM rotenone, and 5 mM antimycin A were prepared in fresh molecular biology-grade DMSO. All stock solutions were aliquoted and stored at −20 °C and diluted in appropriate culture media prior to use. MSO solutions for in vitro studies were prepared fresh in culture media just prior to use. All compounds were purchased from Sigma-Aldrich (St. Louis, MO, USA).

### Immunoblotting

Cell lysates were prepared in RIPA buffer supplemented with protease/phosphatase inhibitor cocktail (Thermo Fisher Scientific, Waltham, MA, USA). Tumor lysates were prepared by grinding frozen tumor tissue with a mortar and pestle and reconstituting in RIPA buffer. Clarified total cellular lysates were immunoblotted with anti-GS, anti-actin (Abcam, Cambridge, MA, USA, ab178422, ab8224), and anti-beta-tubulin (Sigma-Aldrich, T5201) antibodies using standard procedures. For experiments involving proteasome inhibitors, one million glutamine-deprived cells were plated in 10 cm tissue culture plates in media lacking glutamine. The next day, media was replaced with glutamine-containing media with or without MG-132 (10 μM) or Bortezomib (1 μM), as indicated, and lysates were prepared the day after treatment.

### Measurement of oxygen consumption rates

Oxygen consumption rates of live cells were measured in real time using a Seahorse Bioscience XF^e^96 Extracellular Flux Analyzer as previously described^40^. Briefly, cells were plated at 25,000–30,000 cells/well (~80−90% confluent when assayed) in XF96 96-well cell culture plates (Agilent, Santa Clara, CA, USA) and incubated overnight with or without glutamine at 37 °C. Just prior to an assay, growth media was replaced with Seahorse assay media with or without glutamine.

### Generation of stable cell lines

Mission shRNA Lentiviral Transduction Particles (Sigma-Aldrich) were used to generate stable cell lines according to the manufacturer’s instructions. shRNAs targeted human sequences and were as follows: GS sh28 (TRCN0000045628, CCGGGCATCGTGTGT GTGAAGACTTCTCGAGAAGTCTTCACACACACGATGCTTTTTG), GS sh31 (TRCN0000045631, CCGGCCAGGAGAAGAAGGGTTACTTCTCGAGAAGTAAC CCTTCT TCTCCTGGTTTTTG), shControl (SHC004V, TurboGFP shRNA Control Transduction Particles).

### Measurement of cellular proliferation

Cells were plated at 2000–4000 cells/well in 96-well plates. Starting on the day of plating, cellular proliferation was monitored in an IncuCyte FLR Live Cell Analysis System (Essen BioScience, Ann Arbor, MI, USA) in a humidified incubator containing 5% CO_2_ at 37 °C. For proliferation studies involving MSO or nucleoside treatment, cells were treated the day after plating with MSO (1 mM), Gln (2 mM), Glu (4 mM), NH_4_ (0.8 mM), or nucleosides (250 μM) as indicated. For all other proliferation studies, cells were plated in the indicated growth conditions. Nucleosides adenosine, thymidine, guanosine, cytidine, uridine, and inosine were purchased from Sigma-Aldrich and prepared fresh in culture media just prior to use. All proliferation studies were performed at least three times.

### In vivo studies

Animal studies were performed in accordance with the National Institutes of Health Animal Care and Use Committee guidelines. Four- to six-week-old female Fox Chase severe combined immunodeficiency (SCID)-Beige mice were purchased from Charles River Laboratories. Two million cells were suspended in Hank’s Balanced Salt Solution (HBSS, Thermo Fisher Scientific, 14175095) and injected orthotopically into the gastrocnemius muscle in the left hind leg of each mouse. When tumors were palpable, mice were randomized into groups and treated by intraperitoneal injection with either 100 μL of vehicle (0.9% sodium chloride) or MSO (10 mg/kg) once daily, three days per week. Mice were maintained in a pathogen-free environment and monitored by observation of overall health and weekly body weights to determine drug tolerability. Tumors were measured twice weekly with calipers. Tumor volume was calculated by the formula *V*(mm^3^) = (*D* × *d*^2^)/6 × 3.14, where *D* is the longest tumor axis and *d* is the shortest tumor axis.

### Measurement of serum and tumor amino acids

Immediately following euthanasia at study endpoint, blood was collected from mice by intra-cardiac puncture and serum was obtained by centrifugation in BD Microtainer tubes (BD, Franklin Lakes, NJ, USA), and tumors were removed and snap frozen. Samples were stored at −80 °C until processing. To prepare internal standards, stable isotope labeled amino acid standards Glu-d_5_ and Gln-d_5_ (C/D/N Isotopes Inc., Pointe-Claire, Quebec, Canada) were each diluted to 1 mg/ml in ammonium acetate buffer. A 10 µg/ml mixture was made in 90% acetonitrile to be used as the diluent for calibration standards and samples. A 15 µl aliquot of serum was mixed with 150 µl of 90% acetonitrile containing the internal standards. The mixture was centrifuged to pellet proteins and the supernatant was removed to a micro auto-sampler vial for analysis. Tumor specimens were homogenized in 1.0 ml of PBS using a ceramic bead mill. An aliquot equivalent to 2 mg of tissue was transferred to a micro-centrifuge tube and treated in the same manner as serum. Samples were analyzed by high-performance liquid chromatography-mass spectrometry.

### Statistical analysis

Statistical significance was determined by Student *t* test. *p* < 0.05 was considered significant.

## Supplementary information

Supplementary Figures.
