# Supplementary material for: Glutamine synthetase is necessary for sarcoma adaptation to glutamine deprivation and tumor growth
Source: Oncogenesis. 2019 Feb 26;8(3):20. doi: 10.1038/s41389-019-0129-z (PMC6391386; doi:10.1038/s41389-019-0129-z)
Supplement: Supplementary file 1 — Supplementary Figures. [file 41389_2019_129_MOESM1_ESM.pdf]

a

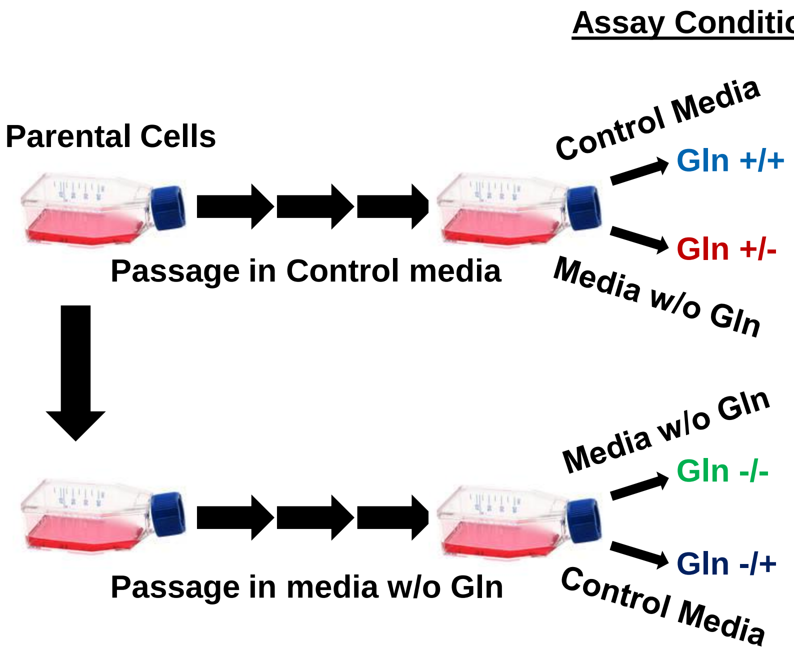

Supplementary Figure S1. Glutamine utilization of human sarcoma cell lines. **a** Schematic of cell line passaging and proliferation assay conditions. **b** Cellular proliferation was monitored in an Incucyte FLR. Cell lines were assayed in RPMI 1640 media supplemented with 10% dialyzed FBS in the presence or absence of glutamine, according to the schematic in A. Gln +/+ (passaged with glutamine, assayed with glutamine), Gln +/- (passaged with glutamine, assayed without glutamine), Gln -/+ (passaged without glutamine, assayed with glutamine), Gln -/- (passaged without glutamine, assayed without glutamine). Data represent the mean ± SD of a representative experiment.

b

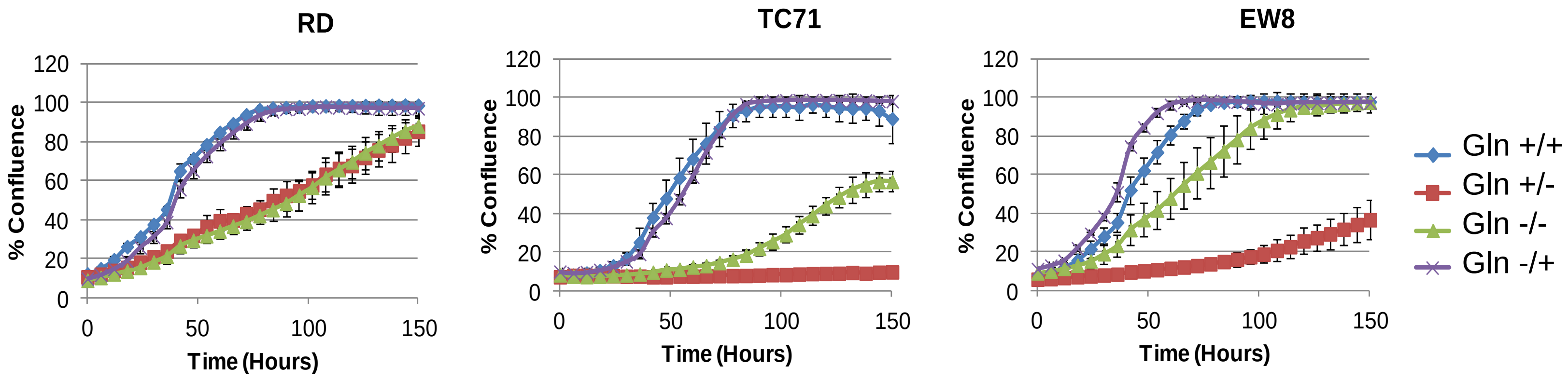

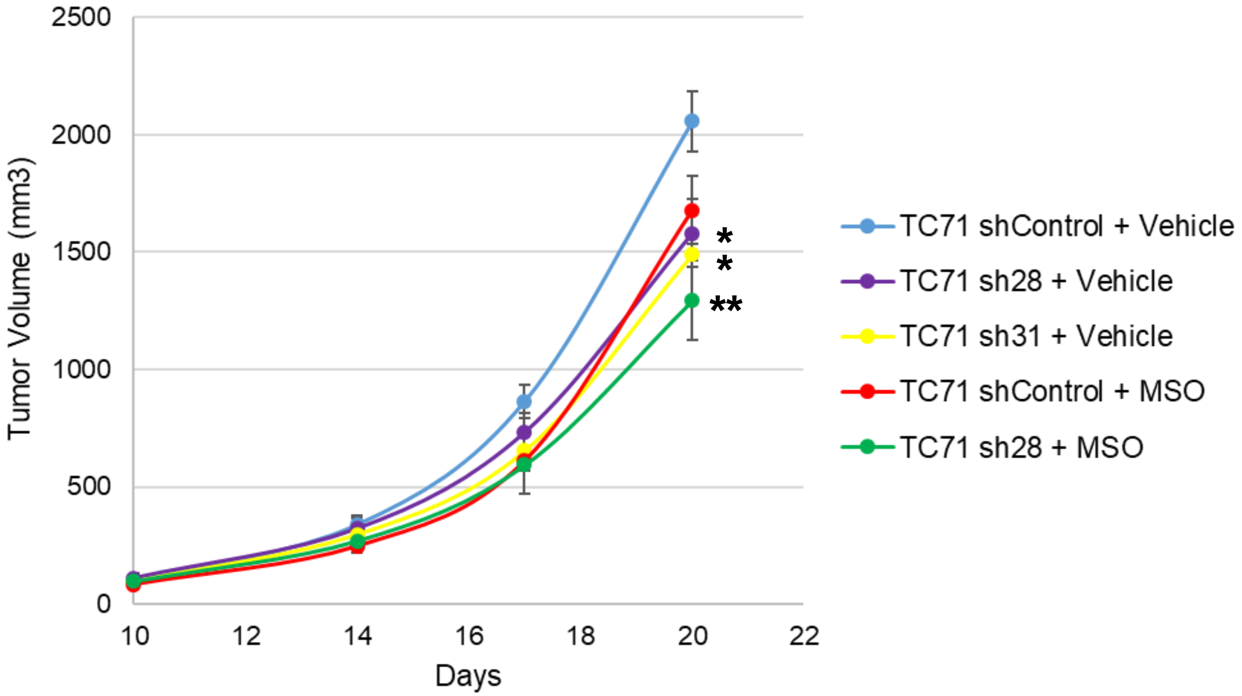

Supplementary Figure S2 Glutamine synthetase inhibition affects sarcoma tumor growth. Average tumor volume over time of mice orthotopically injected with TC71 Ewing sarcoma stable cell lines expressing the indicated shRNAs. Mice were treated with 10 mg/kg MSO or saline (Vehicle), IP, 3 times/week, as indicated. Data represent the mean ± SEM. * $p<0.05$, ** $p<0.005$ by Student *t* test, compared to shControl + Vehicle.

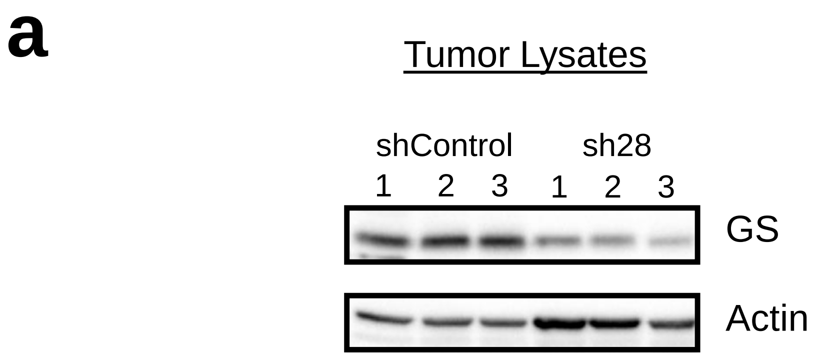

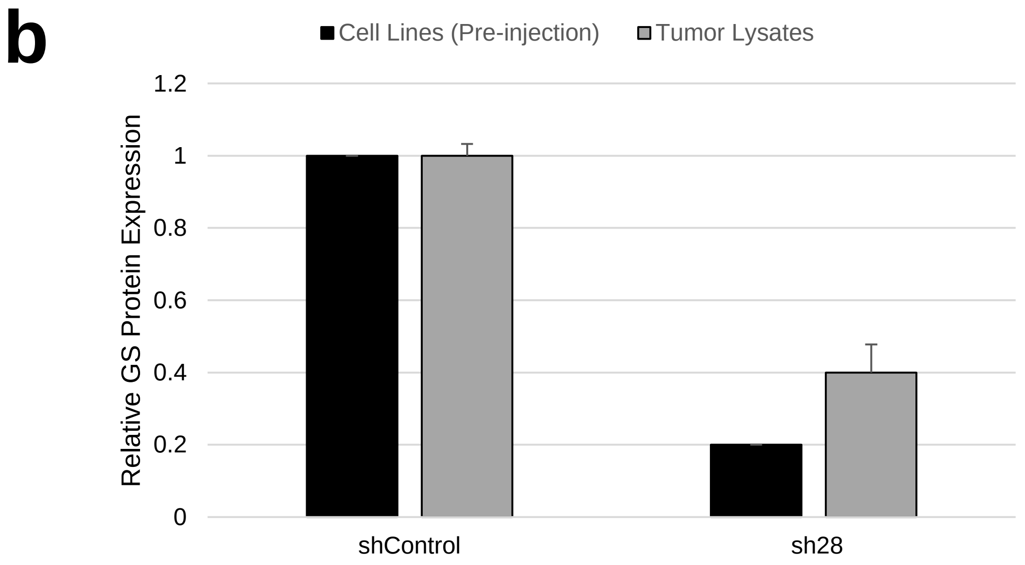

Supplementary Figure S3 Measurement of glutamine synthetase protein expression in tumor lysates**. a** Immunoblot analysis of glutamine synthetase (GS) in tumor lysates derived from cell lines expressing the indicated lentiviral GS shRNAs. Actin was used as a loading control. **b** Quantification of GS protein expression derived from the blots in a (tumor lysates) and Figure 4a (TC71 pre-injection cell lines). Tumor lysate data are the average of the 3 independent mouse tumors shown in a. GS expression is relative to actin and was normalized to shControl. Data represent the mean + SD.

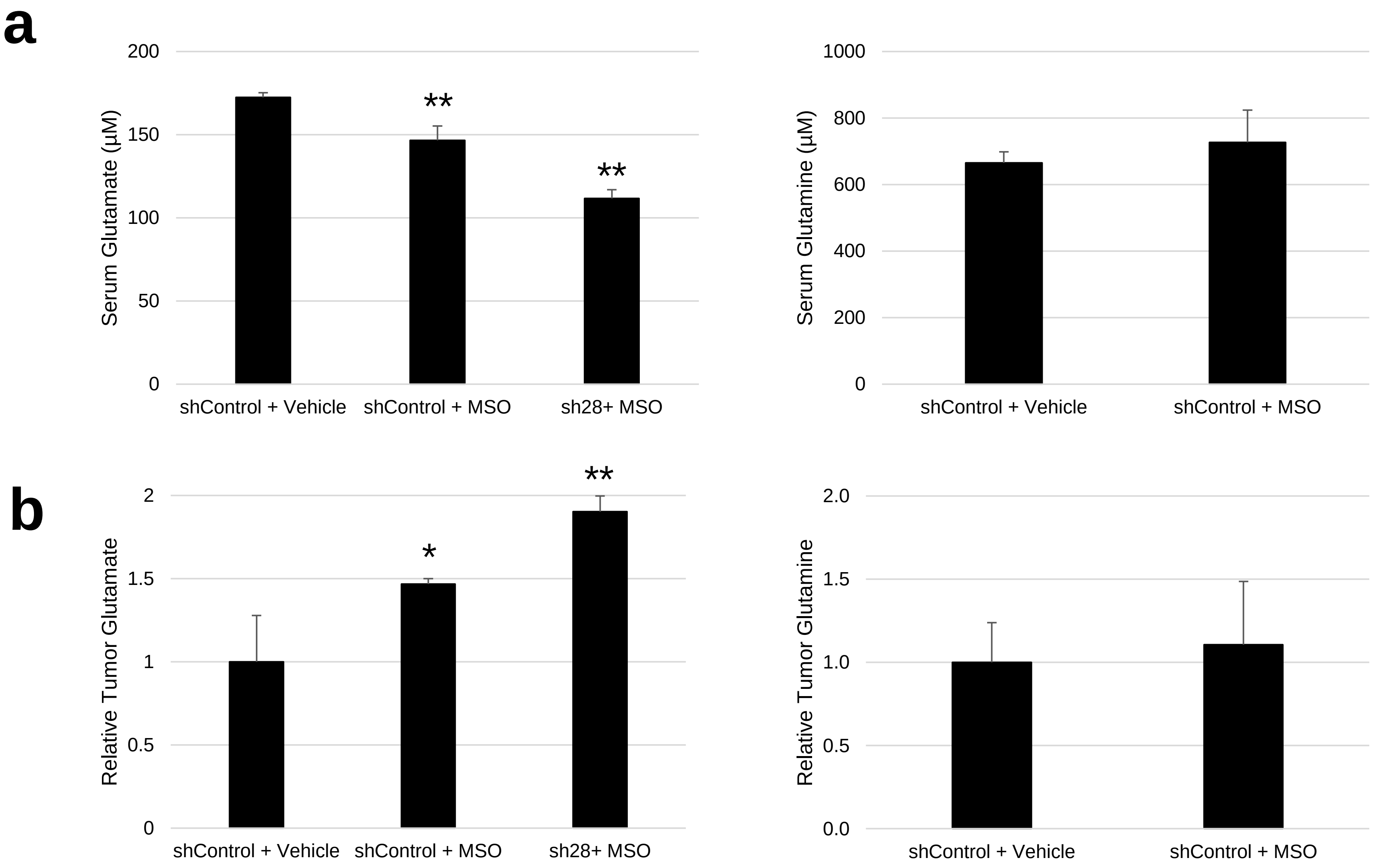

Supplementary Figure S4 Effects of *in vivo* GS inhibition on glutamate and glutamine**.** Glutamate and glutamine were measured in serum (**a**) and tumors (**b**) from mice in the indicated treatment groups. Tumor amino acids were normalized to analyzed tumor specimen weight. Data represent the mean + SD of duplicate analyses of 3 independent mouse samples per condition. * $p<0.05$, ** $p<0.005$ by Student *t* test, compared to shControl + Vehicle.

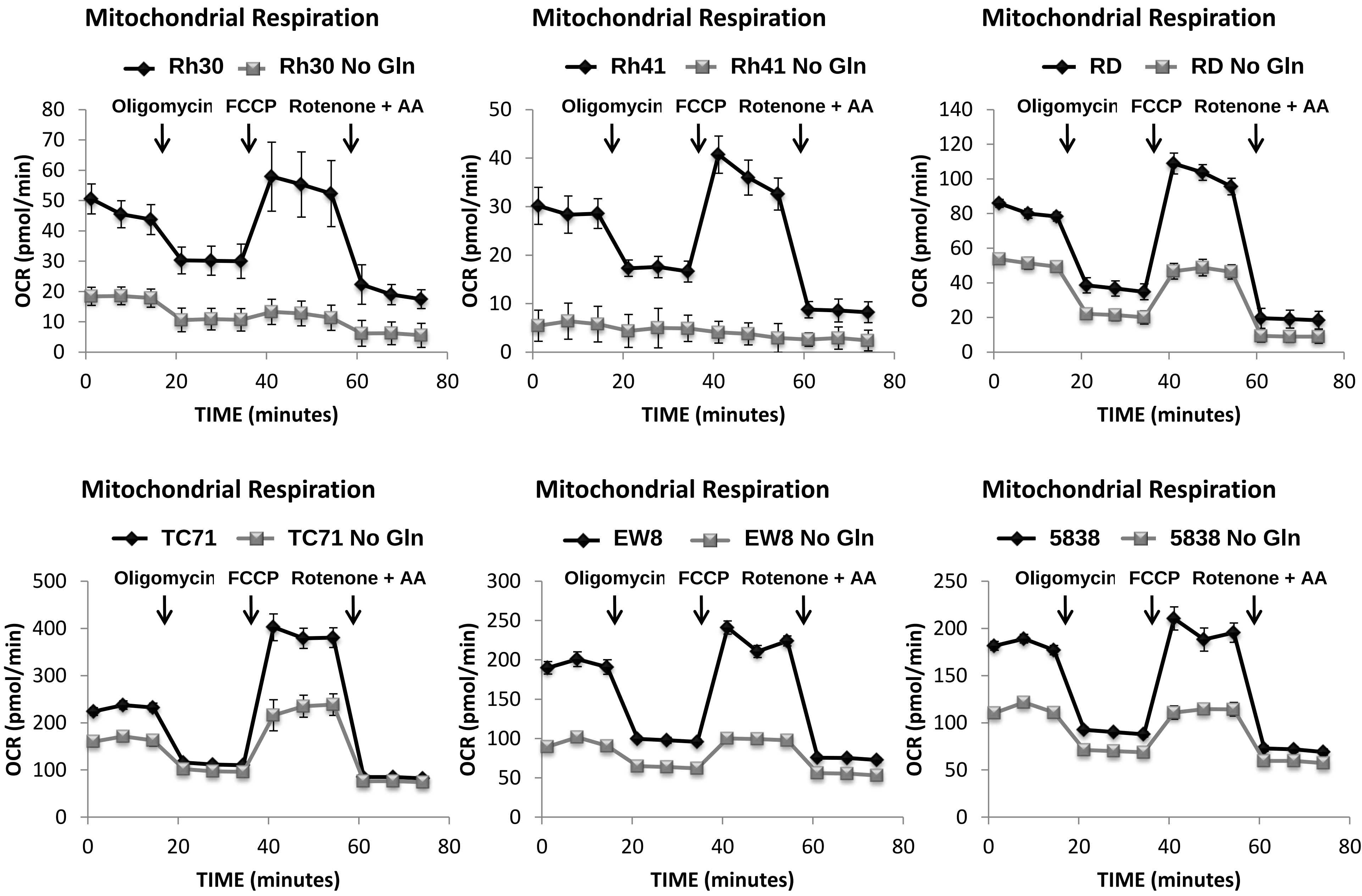

Supplementary Figure S5. Bioenergetic profiles of sarcoma cells grown with or without glutamine. Mitochondrial bioenergetic profiles were generated using the Seahorse XF^{e}96 Analyzer. Data represent the mean ± SD of a representative experiment.
